# Ligand-gated ion channel P2X7 regulates hypoxia-induced factor-1α mediated pain induced by dental pulpitis in the medullary dorsal horn

**DOI:** 10.3389/fnmol.2022.1015751

**Published:** 2022-10-26

**Authors:** Jing Zhang, Jialin Si, Rongrong Liang, Yuxin Lu, Hongwei Shang, Xinwei Li, Shukai Sun, Li-an Wu

**Affiliations:** ^1^State Key Laboratory of Military Stomatology, Department of Pediatric Dentistry, National Clinical Research Center for Oral Diseases, School of Stomatology, The Fourth Military Medical University, Xi’an, China; ^2^College of Life Sciences, Northwest University, Xi’an, China

**Keywords:** P2X7, HIF-1α, pain, dental pulpitis, medullary dorsal horn, microglia

## Abstract

Dental pulpitis often induces severe pain, and the molecular immune response is remarkable in both peripheral and central nervous system. Accumulating evidence indicates that activated microglia in the medullary dorsal horn (MDH) contribute to dental pulpitis induced pain. The P2X7 receptor plays an important role in driving pain and inflammatory processes, and its downstream target hypoxia-induced factor-1α (HIF-1α) has a crucial role in maintaining inflammation. However, the relationship between P2X7 and HIF-1α in dental inflammatory pain remains unclear. This study demonstrated that the degree of inflammation in the dental pulp tissue became more severe in a time-dependent manner by establishing a rat dental pulpitis model *via* pulp exposure. Meanwhile, the expression of P2X7, HIF-1α, IL-1β, and IL-18 in the MDH increased most on the seventh day when the pain threshold was the lowest in the dental pulpitis model. Furthermore, lipopolysaccharides (LPS) increased P2X7-mediated HIF-1α expression in microglia. Notably, the suppression of P2X7 caused less IL-1β and IL-18 release and lower HIF-1α expression, and P2X7 antagonist Brilliant Blue G (BBG) could alleviate pain behaviors of the dental pulpitis rats. In conclusion, our results provide further evidence that P2X7 is a key molecule, which regulates HIF-1α expression and inflammation in dental pulpitis-induced pain.

## Introduction

Dental pulpitis, a dental pulp inflammatory reaction characterized by a high prevalence of pain symptoms, is one of the most common endodontic diseases. During the COVID-19 outbreak, dental pulpitis is the most common disease type among patients undergoing emergency treatment (Salgarello et al., 2021), and dental pulpitis induced pain causes severe discomfort and affects their quality of life. However, the existing treatments strategies and long term efficacy for dental pulpitis are relatively limited. Therefore, it is essential to study the mechanisms underlying the occurrence and development of dental pulpitis-induced inflammatory pain for better clinical treatment.

Previous studies revealed that dental pulp inflammation can lead to pain hypersensitivity, which is strongly associated with increased excitability of the medullary dorsal horn (Dubner and Bennett, 1983; Sessle, 1987; Xie et al., 2007). Moreover, microglia are reported to be involved in the regulation and promotion of the central nervous system (CNS) pain (Inoue and Tsuda, 2018; Tsuda, 2018). Microglia is activated during the dental injuries and inflammation processes (Fan et al., 2010). These findings suggest that microglial activation in MDH is associated with orofacial pain induced by dental pulpitis.

The P2X7 receptor, a member of the P2X receptor family, is widely considered as cationic channel. In addition, as a pro-inflammatory molecule, it regulates many physiological and pathophysiological processes (Dubyak, 2012; Di Virgilio et al., 2017). Further research has shown that the P2X7 receptor is abundantly expressed in the CNS, especially in microglia (Zhou et al., 2010; Calovi et al., 2019). P2X7 participates in pain transmission and the development of inflammation (Liu et al., 2016; Zhang et al., 2020). Itoh et al. tested the P2X7 receptor *in vivo via* an electrophysiological study and found that P2X7 receptors in microglia might be of great importance in central sensitization (Itoh et al., 2011). Therefore, P2X7 in microglia is likely involved in the generation and maintenance of dental pulpitis pain.

Hypoxia-induced factor-1α (HIF-1α), a key transcription factor, is crucial in inflammatory pathways. HIF-1α attenuates myocardial inflammation and may be linked to the NLRP3 inflammasome pathway (Chen et al., 2020). It also reduces inflammation *via* miR-380-3p/NLRP3 (Li et al., 2020). A previous study demonstrated that Brilliant Blue G (BBG), as a selective and non-competitive P2X7 antagonist, can prevent the activation of NLRP3 inflammasome and participate in the regulation of pain sensitization (D'Amico et al., 2021). It has also been reported that persistent upregulation of HIF-1α is mediated by P2X7 (Hirayama and Koizumi, 2017). Additionally, HIF-1α is involved in neuropathic pain in a chronic constriction injury model (Hsieh et al., 2012). However, the relationship between P2X7 and HIF-1α in dental pulpitis induced pain remains unclear.

This study aimed to verify the relationship between P2X7/HIF-1α and dental pulp inflammatory pain and to test changes in inflammatory factors in the dental pulpitis model. Collectively, we found that P2X7-mediated HIF-1α expression in microglia in the MDH is of great importance in the generation and maintenance of pain in dental pulpitis.

## Materials and methods

### Animals

All processes were examined and approved by the Animal Use and Care Committee of the Fourth Military Medical University (SYXK2008-005). Male Sprague–Dawley rats (200–250 g) were obtained from the Animal Center of the Fourth Military Medical University. Up to six rats were placed per cage at 23°C with 50% humidity and a controlled 12-h light/dark cycle. Sufficient water and food were provided to the rats. The rats in the experimental group underwent tooth pulp exposure (exposure time length: 1, 3, 7, and 14 days). Sham-operated rats served as the controls.

### Dental pulp exposure procedure

The model in this study was established using the dental pulp exposure procedure as described previously (Sun et al., 2019). The rats were grouped into the following subsets according to the pulp exposure time: 1, 3, 7, and 14 days. First, we doped the rats with pentobarbital (50 mg/kg) *via* intraperitoneal injection and then fixed them on an operating board. The rats were positioned face up. The mouths of the rats were opened using retractors. The pulp of the maxillary left first molar was exposed using a rotated ball-shaped dental bur, without additional operations. Subsequently, the rats were again placed in their cages. In the control group, rats were anesthetized without pulp exposure.

### Drug administration and experimental design

Eighteen rats were randomly divided into sham, vehicle, and BBG groups. The sham rats were treated with saline intraperitoneally (i.p.) every other day for 7 days. The vehicle rats were subjected to injection of saline (i.p.) every other day after the dental pulp exposure for 7 days. BBG (Sigma-Aldrich, United States), a selective and non-competitive P2X7 antagonist, was administrated in 50 mg/kg to rats every other day (i.p.) after the dental pulp exposure for 7 days. The dose was chosen based on previous studies (Guo et al., 2020; D'Amico et al., 2021).

### Behavioral test

Behavioral tests were performed by measuring the facial skin withdrawal threshold (WT) with von Frey filaments (Danmic Global, United States). Values of von Frey filaments were: 3.84, 4.08, 4.17, 4.31, 4.56, 4.74, 4.93, 5.07, 5.18, 5.46, and 5.88. Before testing, the rats were placed into a small porous metal mesh cage (6 cm × 6 cm × 20 cm) for 30 min to adapt to the testing environment. Then, we applied von Frey filaments to prick the facial skin of rats when their heads were in a resting and steady position. Each filament was applied 10 times; with pause at least 5 s between the two applications. Rats were stimulated again by the next filament after a 3-min rest. The minimum filament value causing over five positive responses (rapid retraction, biting, and multiple head-shaking within 5 s in each stimulation) in 10 tests was taken as the pain threshold.

### Haematoxylin and eosin staining

The whole maxillary dentitions of rats were taken out and the left maxillary first molars were instantly fixed in 4% paraformaldehyde (PFA) for 1 day, and decalcified with 10% ethylene diamine tetraacetic acid (EDTA) for 6 weeks. Then, the teeth were embedded in paraffin and sliced at 5 μm. The sections were dewaxed, stained by hematoxylin and eosin. After dehydration, sections were sealed with neutral gum. An ordinary microscope was used to observe and photograph the images.

### Immunofluorescence staining

The rats were anesthetized with pentobarbital (50 mg/kg) and fixed *via* heart perfusion. When the perfusion finished, brains were removed charily, and post-fixed for 8 h in 4% PFA, and treated with 30% sucrose at 4°C until the tissue sank and reached the bottom. Tissue slices (25 μm) were cut using a freezing microtome. The slices were then cleaned three times with Phosphate buffered saline (PBS) and blocked with 10% donkey serum for 1 h. After that, the sections were blocked for over 8 h at 4°C with primary antibodies: mouse anti-P2X7 (1:100; Santa Cruz), goat anti-Iba-1 (1:400; Abcam), rabbit anti-GFAP(1:500; Abcam), rabbit anti-NeuN(1:500; Abcam), and rabbit anti-HIF-1α (1:200; Affinity). After three washes with PBS, the sections were further stained with Alexa Fluor secondary antibodies such as donkey-anti-goat or rabbit IgG Alexa Fluor 594 (1:1,000; Abbkine), and donkey-anti mouse IgG Alexa Fluor 488 (1:1,000; Abbkine) for 2 h at room temperature. Subsequently, a laser scanning confocal microscope (Olympus FV1000, Japan) was used to photograph the images.

### Western blotting

Left MDH tissues were collected on ice from anesthetized rat brains. An ultrasonic homogenizer was used to homogenize MDH tissues in Radioimmunoprecipitation assay (RIPA) lysis buffer (Beyotime Biotechnology, China). The protein concentration of the samples was measured by the bicinchoninic acid method using the Pierce™ rapid gold BCA protein assay kit (Thermo Fisher Scientific, United States). Proteins were separated by electrophoresis on a 4% stacking/10% separating polyacrylamide gel (Bio-Rad, United States). A PVDF film (Immobilon-P, Millipore, United States) was then used to transfer the proteins. The membrane blots were washed with PBS every 10 min for half an hour. Membranes were blocked with 5% BSA for 2 h and rewashed. The membranes were incubated overnight at 4°C with the following primary antibodies: mouse anti-HIF-1α (1:400; Affinity), mouse anti-β-actin (1:1,000; Santa Cruz), mouse anti-P2X7 (1:500; Santa Cruz), rabbit anti-IL-1β (1:1,000; Abcam), and rabbit anti-IL-18 (1:1,000; Abcam). On the second day, the membranes were cleaned with TBS-T and then hatched with HRP-conjugated secondary antibodies against mouse or rabbit (1:3,000; GeneTex) for 60 min at room temperature. The membranes were washed three times with TBS-T. Finally, the images of proteins were acquired by enhanced chemiluminescence (ECL) and exposed to a film.

### Quantitative real-time PCR

Whole RNA was extracted from the left MDH of rats using TRIzol reagent (Thermo Fisher Scientific, United States), and the PrimeScript RT Master Mix (TaKaRa, Japan) was used for cDNA synthesis. The cDNA was stored at −20°C after reaction for 15 min at 42°C and 15 s at 85°C. Quantitative PCR was performed on an ABI 7500 Detection System (Applied Biosystems, United States) using SYBR® Green MasterMix. The conditions of PCR were 95°C for 60 s, 95°C for 5 s, 60°C for 20 s, and 95°C for 15 s for 45 cycles. The relative mRNA levels were analyzed, and all results were standardized to β-actin. The primer sequences used for qRT-PCR were synthesized by Tsingke Biotechnology (shown in Table 1).

**Table 1:** Sequences of the primers for Quantitative real-time PCR.

Gene	Primer type	Sequence 5′-3′
P2X7	Forward	CCCATCTATGAGGGTTACGC
P2X7	Reverse	TTTAATGTCACGCACGATTTC
HIF-1α	Forward	AACAGCCAATGAGTCCGAGG
HIF-1α	Reverse	TTTGAAGGTGTAGGGACGGC
IL-1β	Forward	TGTCTGAAGCAGCTATGGCA
IL-1β	Reverse	GAACAGGTCATTCTCCTCACTGT
IL-18	Forward	TCAGCTCTTCTACCAGCAAACA
IL-18	Reverse	TTCCAACTGAGAGGCTGTGC
β-actin	Forward	CAAGCAGCAGGAATTGGAACG
β-actin	Reverse	CTCATCCATTGACTGCCCCA

### Cells culture and siRNA transfection

BV2 cells were used as the microglial cell line in this experiment. Cells were cultured in Dulbecco’s Modified Eagle Medium (DMEM), supplemented with 10% Fetal Bovine Serum (FBS; Gibco, United States) as well as 1% penicillin/streptomycin, and cells were incubated at 37°C with 5% CO_2_. In accordance with the manufacturer’s protocol, BV2 cells were transfected with siRNA oligos targeting P2X7 or negative control siRNA (GenePharma, China), using Lipofectamine 2000 (Invitrogen, United States). The cells were collected for western blotting after 48 h.

### Statistical analysis

All data are presented as mean ± SD. The results and statistical graphs were analyzed using GraphPad Prism 8.0. The Student’s *t*-test was used to analyze the statistical differences between the two groups. Multiple group comparisons were determined by one-way ANOVA with Tukey’s test for *post hoc* analysis. Pearson correlation analysis was used for two-variable correlation analysis and was calculated by the Coloc 2 plug-in of Image J software. Statistical significance was expressed as a *p* < 0.05.

## Results

### Establishment of the rat dental pulpitis pain model

**Figure 1:**
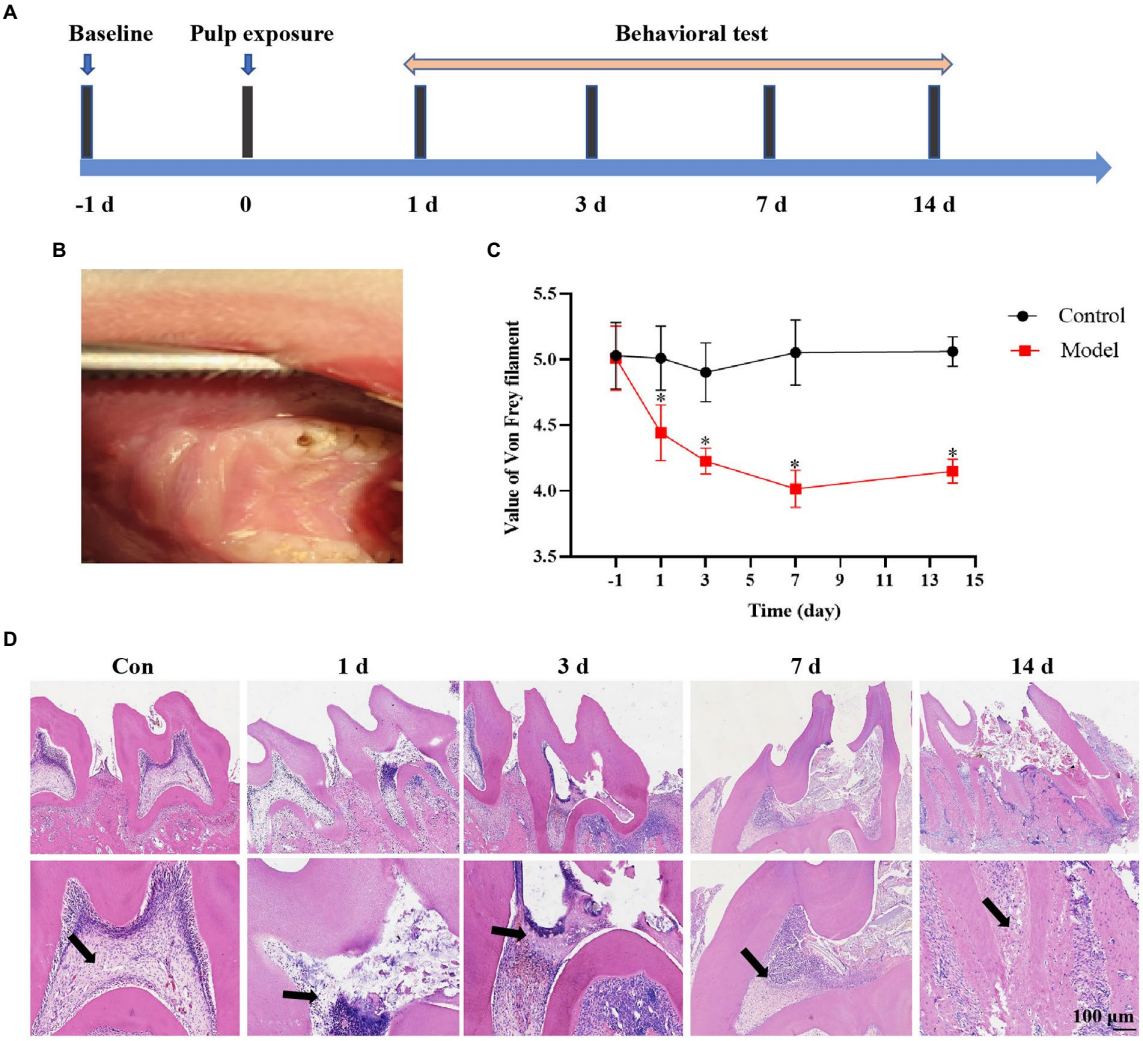
Establishment of the rat dental pulpitis pain model. **(A)** Schedule of pulp exposure treatment and behavioral test. **(B)** A rat dental pulpitis model was established *via* dental pulp exposure. **(C)** The pain threshold decreased with a prolonged exposure time of the dental pulp, reaching the minimum on the seventh day, and then increasing slightly during the next 7 days. *p < 0.05 vs. control. **(D)** Haematoxylin and eosin (H&E) staining showed that the inflammatory infiltration range in dental pulpitis tissue gradually expanded from the crown to the root pulp as the exposure time prolonged. Scale bar = 100 μm.

We established a rat pulpitis pain model by exposuring the dental pulp and behavioral tests were then detected prior to dental pulp exposure or 1, 3, 7, and 14 days (Figures 1A,B). The behavioral results showed that the pain threshold decreased with a prolonged exposure time of the dental pulp in the dental pulpitis model compared with that in the control group and reached the minimum on the seventh day (Figure 1C). Haematoxylin and eosin (H&E) staining results showed advances in inflammatory infiltration in the dental pulp tissue. No inflammatory cell infiltration was observed in the control group. The pulp inflammation range in the dental pulpitis group gradually expanded from the crown to the root pulp as the modeling time was extended (Figure 1D). These data suggest that a more severe inflammatory response results in a lower mechanical pain threshold in rats with dental pulpitis.

### Activation of microglia after dental pulp exposure caused the release of inflammatory factors in the medullary dorsal horn

**Figure 2:**
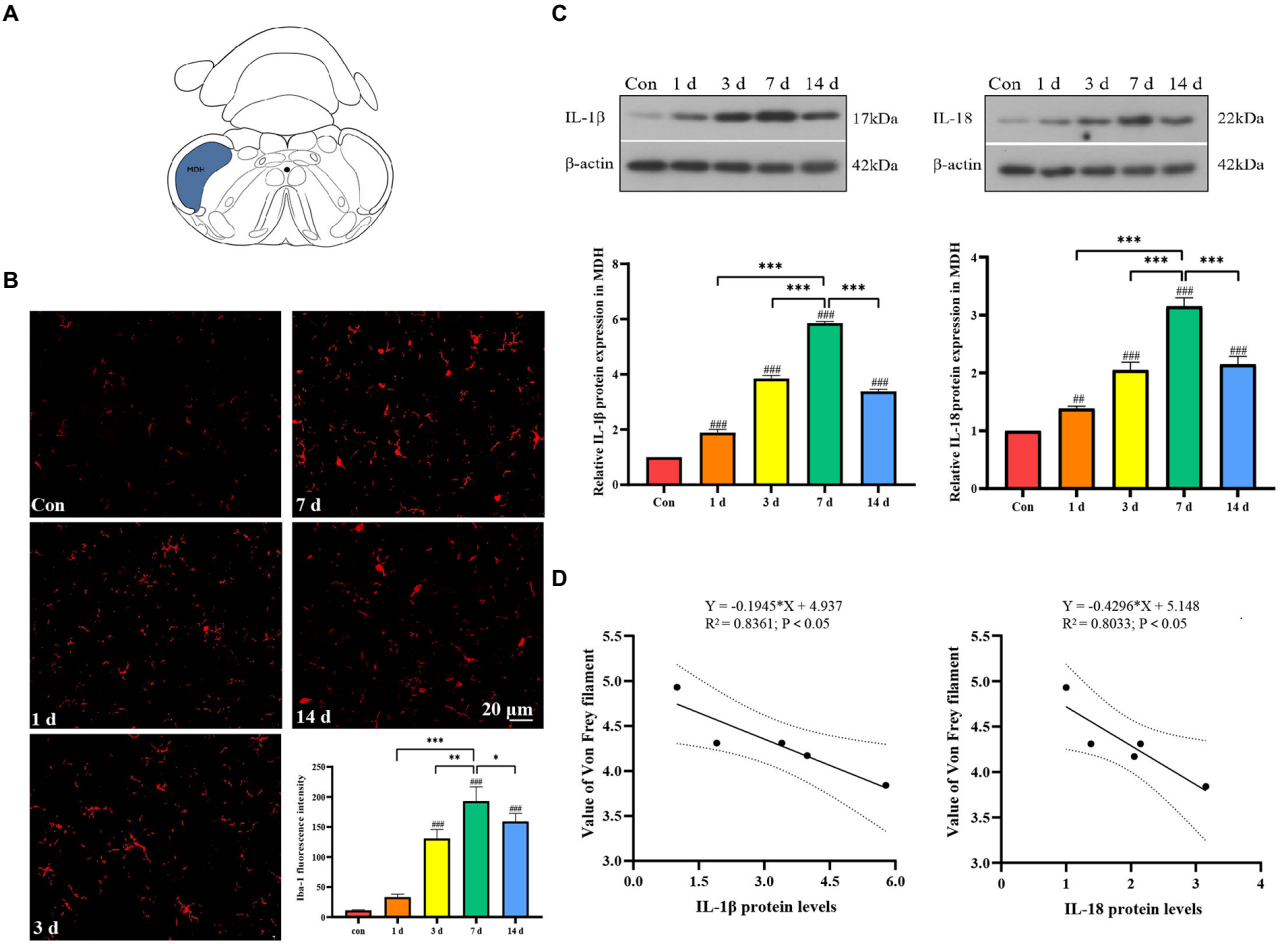
Activation of microglia and release of inflammatory factors in medullary dorsal horn (MDH) after dental pulp exposure. **(A)** The location of the MDH in the representative coronal brain slices schematic diagram. **(B)** The expression of Iba-1 after dental pulp exposure. **(C)** The protein levels of IL-1β and IL-18 in MDH. **(D)** Pearson correlation analysis of the withdrawal threshold and IL-1β and IL-18 protein levels in MDH. *p < 0.05, **p < 0.01 vs. 7 d group, ^***^p < 0.001 vs. 7 days group, ^##^*p* < 0.01, ^###^*p* < 0.001 vs. control.

Microglia in left MDH were activated after dental pulp exposure, and the expression of the Iba-1 was significantly increased，especially on the seventh day. We could observe that the activated Iba-1 positive cell had a larger cell body with shorter protuberances, while the resting microglia had more spindly branches (Figures 2A,B). Western blot results revealed that IL-1β and IL-18 in the MDH of the dental pulpitis model increased from 1 to 14 days compared to the control, reaching its peak on the seventh day, and then decreased slightly on the fourteenth day (Figure 2C). Interestingly, the negative correlation between the withdrawal threshold and the levels of IL-1β and IL-18 seems to be obvious (Figure 2D).

### Increased expression of P2X7 receptor in the microglia of medullary dorsal horn from dental pulpitis model

**Figure 3:**
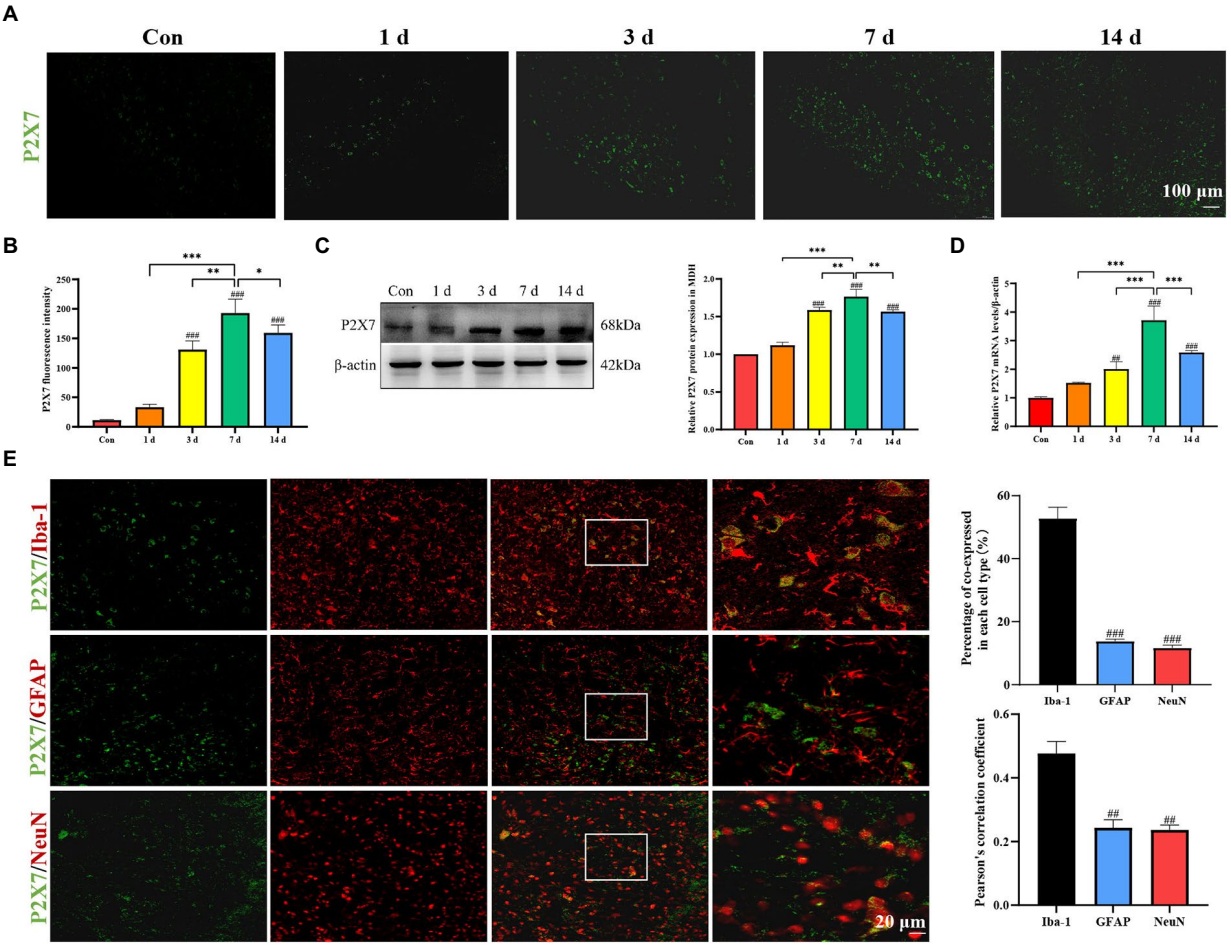
Expression of P2X7 in MDH after dental pulp exposure. **(A,B)** Immunofluorescence staining showed that expression of P2X7 in MDH gradually increased with a prolonged exposure time of the dental pulp. **(C)** Western blot analysis showed that P2X7 protein levels markedly increased compared to the control group, with the highest expression on the seventh day. **(D)** The mRNA levels of P2X7 increased gradually with a prolonged pulp exposure time with the highest expression on the seventh day. ^*^p < 0.05, ^**^p < 0.01, ^***^p < 0.001 vs. 7 days group, ^##^*p* < 0.01, ^###^*p* < 0.001 vs. control. **(E)** Double immunofluorescence staining showed that P2X7 was mainly located in microglia, rather than in astrocytes nor neurons. ^##^*p* < 0.01, ^###^*p* < 0.001 vs. Iba-1 group.

Immunofluorescence staining results showed a significant increase of P2X7 expression in the MDH of dental pulpitis model compared to the control group (Figures 3A,B). Western blot analysis demonstrated that the protein levels of P2X7 changed over time (Figure 3C). In addition, the mRNA levels of P2X7 were markedly increased with the highest expression on the seventh day (Figure 3D). To reveal the location of P2X7, we used several specific markers, including Iba-1 for microglia, GFAP for astrocytes, and NeuN for neurons, to analyze their co-expression with P2X7. As shown in Figure 3E, double-immunofluorescence staining showed that P2X7 was mainly colocalized with Iba-1, with minor colocalizations with GFAP and NeuN (Figure 3E). Therefore, the above results suggest that P2X7 expression in the microglia of the MDH was upregulated by dental pulp exposure.

### Upregulation of HIF-1α expression in dental pulpitis model

**Figure 4:**
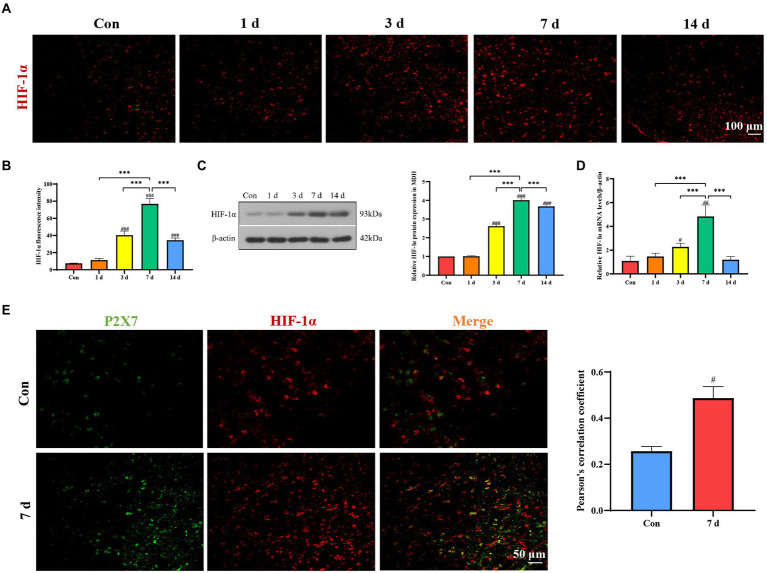
Expression of HIF-1α in MDH after dental pulp exposure. **(A,B)** Immunofluorescence staining showed that expression of HIF-1α in MDH gradually increased with a prolonged exposure time of the dental pulp. **(C)** Western blot analysis showed that HIF-1α protein levels significantly increased compared to the control group, with the highest expression on the seventh day. **(D)** The mRNA levels of HIF-1α increased with the highest expression on the seventh day. **(E)** Double immunofluorescence staining revealed that P2X7 is colocalized with HIF-1α in MDH. ^***^p < 0.001 vs. 7 days group, ^#^*p* < 0.05, ^##^*p* < 0 0.01, and ^###^*p* < 0.001 vs. control.

Immunofluorescence staining, Western blotting, and qRT-PCR were used for detection of HIF-1α expression in dental pulpitis model. The results revealed that the expression of HIF-1α gradually increased and reached the peak on the seventh day (Figures 4A–D). Pearson’s correlation coefficient is the most commonly used quantitative description of the colocalization and has been used to manifest the co-expression changes as described previously (Guo et al., 2020; Cheng et al., 2021; Yang et al., 2021). Double immunofluorescence staining demonstrated that P2X7 was co-expressed with HIF-1α, and its expression was increased compared to that in the control group (Figure 4E).

### Inhibition of P2X7 decreased LPS-induced HIF-1α expression and inflammatory factors levels in BV2 cell lines

**Figure 5:**
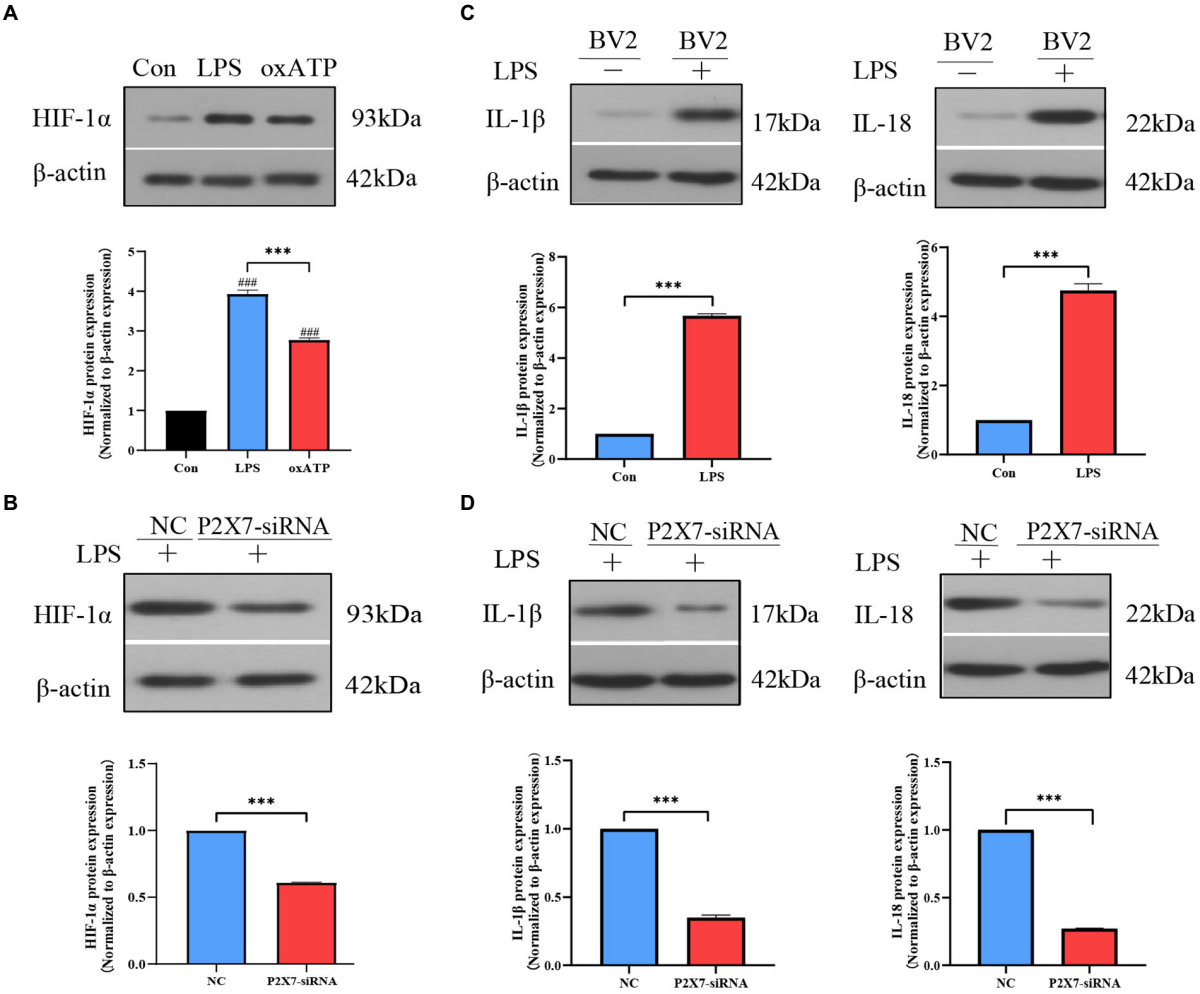
Effects of P2X7 on HIF-1α expression and inflammatory factors levels in BV2 cells. **(A)** Western blot results showed that LPS-induced upregulation of HIF-1α expression which was inhibited by oxATP. ^###^*p* < 0.001 vs. control, ***p < 0.001 vs. LPS group. **(B)** HIF-1α expression was downregulated with P2X7 siRNA pretreatment. ^***^p < 0.001 vs. negative control group. **(C)** LPS induced upregulation of IL-1β and IL-18. ^***^p < 0.001 vs. control. **(D)** The protein expression levels of IL-1β and IL-18 in BV2 cells treated with siRNA against P2X7. ^***^p < 0.001 vs. negative control group.

We simulated an inflammatory environment *in vitro*, a marked increase of HIF-1α expression levels was observed after LPS (1 μg/ml) stimulation, but the P2X7 antagonist oxidized ATP (oxATP) inhibited the increase of LPS-induced HIF-1α expression (Figure 5A). To investigate the effect of P2X7 on HIF-1α and inflammatory factors, RNA interference experiments targeting P2X7 were performed in BV2 cells. The knockdown efficiency of P2X7 siRNA was tested by western blot and qRT-PCR, we could observe the P2X7 expression was inhibited after treatment with P2X7 siRNA (Supplementary Figure 1). We also found that HIF-1α expression in P2X7-siRNA-treated BV2 cells was lower than that in the negative control group (Figure 5B). Furthermore, the protein expression levels of IL-1β and IL-18 were markedly increased after LPS treatment (Figure 5C). However, the protein levels of IL-1β and IL-18 were lower in BV2 cells treated with LPS and siRNA against P2X7, as compared to BV2 cells stimulated with LPS only (Figure 5D).

### Inhibition of P2X7 by BBG alleviated the pain behaviors of dental pulpitis rats and decreased the expressions of HIF-1α and inflammatory factors

**Figure 6:**
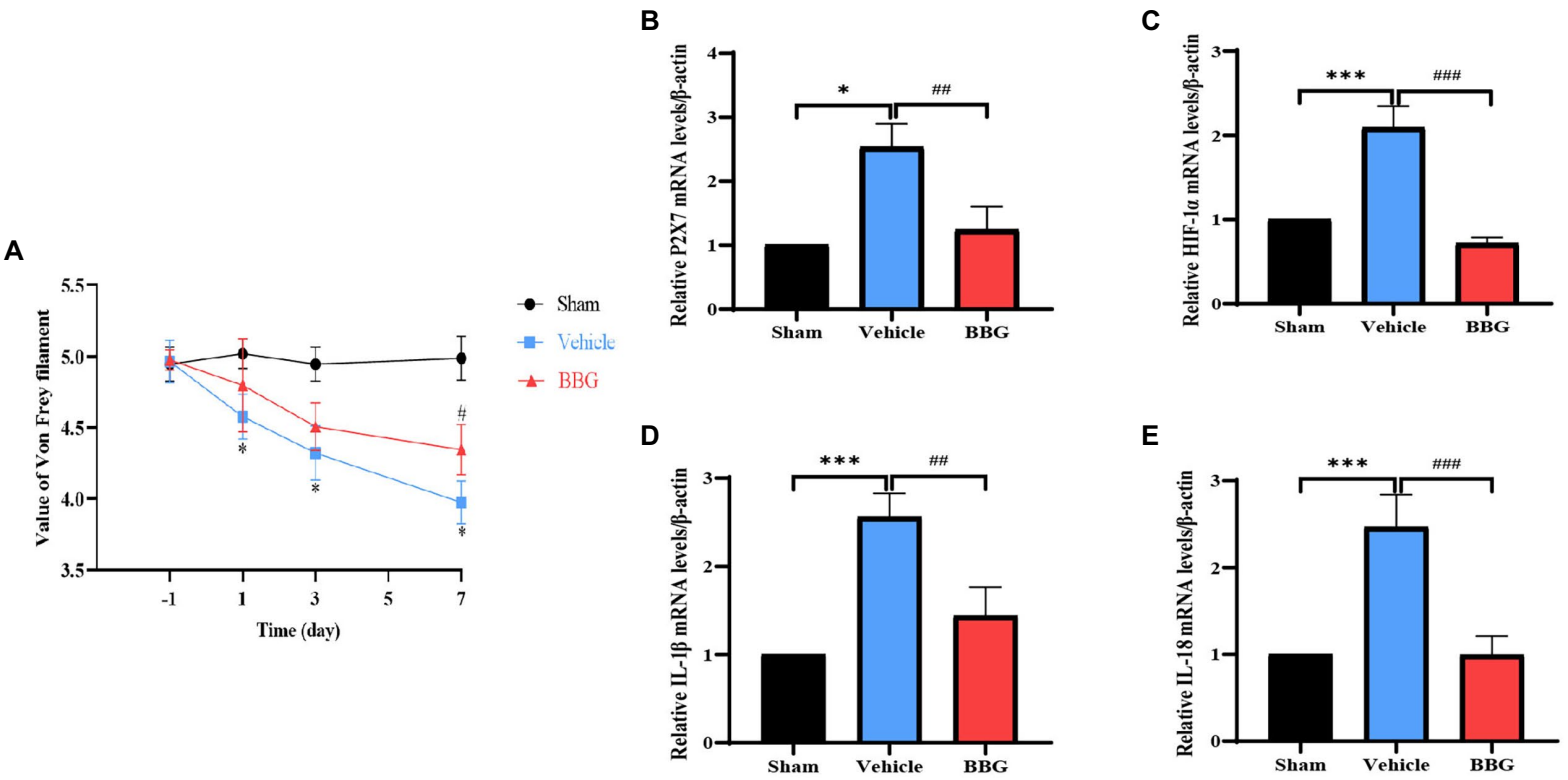
Effects of Brilliant Blue G (BBG) on pain behaviors and the expression of P2X7, HIF-1α, and inflammatory factors. **(A)** The pain threshold of the sham, vehicle and BBG rats. ^*^p < 0.05 vs. sham, ^#^*p* < 0.05 vs. vehicle. **(B)** The effect of dental pulp exposure and BBG treatment on mRNA level of P2X7 in MDH. *p < 0.05 vs. sham, ^##^*p* < 0.01 vs. vehicle. **(C)** The effect of dental pulp exposure and BBG treatment on mRNA level of HIF-1α in MDH. ***p < 0.001 vs. sham, ^###^*p* < 0.01 vs. vehicle. **(D)** The effect of dental pulp exposure and BBG treatment on mRNA level of IL-1β in MDH. ^***^p < 0.01 vs. sham, ^##^*p* < 0.01 vs. vehicle. **(E)** The effect of dental pulp exposure and BBG treatment on mRNA level of IL-18 in MDH. ^***^p < 0.001 vs. sham, ^###^*p* < 0.01 vs. vehicle.

To verify whether the pain behaviors would be alleviated after P2X7 blockade in the rat dental pulpitis model, the rats were administrated by BBG. Behavioral tests were performed by von Frey filaments, and the results showed that the pain threshold of the vehicle rats decreased compared to the sham rats. Notably, after injection of BBG, the pain threshold significantly increased compared to the vehicle rats. The results indicate that BBG could alleviate the dental pulpitis-induced pain behaviors of rats (Figure 6A). The qRT-PCR results showed that BBG could significantly decrease the expression of P2X7, compared with the vehicle group (Figure 6B). Moreover, as a selective and non-competitive P2X7 antagonist, BBG also reduced the expression of HIF-1α compared with the vehicle rats (Figure 6C). These results suggest that HIF-1α is mediated by P2X7. Additionally, the expression changes of inflammatory cytokines in MDH were also tested by qRT-PCR after injection of BBG on the seventh day. We observed that administration of BBG significantly reduced the mRNA levels of IL-1β and IL-18 in MDH compared with the vehicle rats (Figures 6D,E).

## Discussion

Pain symptoms are the most frequently experienced orofacial complaints in patients with dental pulpitis (Lipton et al., 1993). Bacterial infections are the primary cause of dental pulpitis. In response to infection and inflammation, glial cells are reactivated and release mediators, including cytokines and chemokines, which can induce can induce peripheral and central sensitization. Notably, regulating inflammation and the glial state has recently been demonstrated to promote pulp inflammation-related pain relief and may become a potential therapeutic target (Haug and Marthinussen, 2019; Ye et al., 2021).

In the present study, we established a rat dental pulpitis model and found that the pain threshold decreased and reached the minimum on the seventh day. The range of inflammation in dental pulp tissue increased with prolonged dental pulp exposure time. Furthermore, microglias in the MDH were activated in the rat dental pulpitis model. These results collectively indicate that the activation of microglia in the MDH is important in the development of dental pulpitis, consistent with findings in other dental nociceptive models (Xie et al., 2007; Fan et al., 2010).

The P2X7 receptor has drawn much attention because of its special role in driving inflammatory processes and pathological pain in nervous system diseases (Takenouchi et al., 2010; Yang et al., 2015; Cheffer et al., 2018; Yin et al., 2021). Notably, studies have shown that upregulation of P2X7 contributes to microglial activation (He et al., 2012; Zhou et al., 2019; Yin et al., 2021). Our present study confirmed that dental pulpitis pain can activate the expression of P2X7 in microglia in the MDH, with the highest expression on the seventh day, which coincided with the most severe pain symptoms. Although much progress has been made in recent years to understand the role of P2X7 in central sensitization and pain sensation, more work is still required to elucidate the underlying mechanisms, especially in dental pulpitis pain.

Hypoxia-induced factor-1α is a transcription factor that controls the expression of key proteins during hypoxia, ischemia, and inflammation (Fraisl et al., 2009). Infiltrated inflammatory cells led to the accumulation of HIF-1α with increased TNF-α and IL-1β (Dehne and Brune, 2009; Li et al., 2011; Sasaki et al., 2019). It has been reported that HIF-1α promotes the expression of IL-1β and TNF-α in inflammatory dental pulp cells, and HIF-1α is involved in the development of dental pulpitis from reversible to irreversible pulpitis (Fujii et al., 2020). However, the function of HIF-1α in the MDH during the pain caused by dental pulpitis is still unclear. In this study, we found that HIF-1α was gradually increased in the MDH, and the expression trend was consistent with P2X7, reaching a peak after 7 days of dental pulp exposure.

To further verify the relationship between P2X7 and HIF-1α in dental pulpitis induced pain, we used BBG to blockade P2X7 in the rat dental pulpitis pain model. Our results confirm the suppressed effects of BBG on P2X7, simultaneously; BBG reduced the expression of HIF-1α, which suggests P2X7-mediated HIF-1α expression in dental pulpitis induced pain. Additionally, experiments *in vitro* were performed in a simulated inflammatory environment. LPS stimulation was used for P2X7 receptor activation, and oxATP, an antagonist of the P2X7 receptor, was applied to inhibit P2X7 receptor activation. Our results demonstrated that LPS can upregulate the expression of HIF-1α, while oxATP decreased upregulation of HIF-1α stimulated by LPS. Besides, after treatment with P2X7-siRNA, similar results were obtained. In BV2 cells, siRNA-mediated downregulation of P2X7 significantly reduced HIF-1α expression. These findings also suggest that the association between P2X7 and HIF-1α in microglia contributes to LPS-induced inflammation. This provides a theoretical foundation for P2X7 as a treatment target for dental pulp inflammation-induced pain. Interestingly, Hirayama et al. found that HIF-1α is dependent on P2X7 receptor-mediated purinergic signaling in astrocytes. Stimulation of astrocytes with the P2X7 receptor agonist BzATP could induce the upregulation of HIF-1α, but BzATP failed to increase HIF-1α in neurons (Hirayama and Koizumi, 2017). These results support the notion that in astrocytes, HIF-1α is not hypoxia-dependent but rather P2X7 receptor-dependent. Our study showed similar findings in microglia of MDH, but not astrocytes nor neurons. Moreover, one study showed that HIF-1α protein expression increased with BBG treatment in tumor cells (Fang et al., 2013). Therefore, the underlying mechanisms of P2X7-mediated HIF-1α expression in microglia of MDH induced by dental pulpitis need to be further explored.

Accumulating evidence has confirmed that proinflammatory cytokines are released from immune cells and glia (Ferrari et al., 1997; Chakfe et al., 2002; Rampe et al., 2004). The expression level of proinflammatory cytokines indicates activation of microglia and severity of inflammation. To determine whether the dental pulpitis induced pain is associated with the expression of inflammatory markers in the MDH, we chose IL-1β and IL-18 as testing metrics. Our results showed that in the rat dental pulpitis model, IL-1β and IL-18 levels increased in a similar manner, and the expression peak occurred on the seventh day, which was contrary to the changes of the pain threshold. Similarly, Haug et al., in their study of 42 patients who experienced dental pain, IL-1β and IL-6 levels were higher than those in the control group (Haug and Marthinussen, 2019). Our experimental results also demonstrated that the injection of BBG could reverse the pain threshold and alleviate dental pulpitis induced pain through downregulated expression of IL-1β and IL-18 *via* inhibiting HIF-1α. Concurrently, the results *in vitro* showed that treatment with LPS increased IL-1β and IL-18, but downregulation of P2X7 caused a decrease in IL-1β and IL-18, which might be related to the decrease of HIF-1α expression. It has been proved that accumulated HIF-1α promotes the release of inflammatory factors, leading to microglial activation (Yuan et al., 2021). Additionally, HIF-1α may regulate the activation of the NLRP3 inflammasome *via* NF-κB and promote the expression of IL-1β (Huang et al., 2019). This implies the association of P2X7 with HIF-1α in microglia could mediate inflammatory cytokine production. Previous studies have confirmed that enhanced expression of proinflammatory factors was involved in central sensitization, which could promote inflammatory pain (Kiyomoto et al., 2015; Boucher et al., 2018; Yamashita et al., 2021).

In summary, our study revealed a positive correlation between the expression levels of P2X7/HIF-1α in microglia of MDH and the degree of dental pain induced by pulpitis. Furthermore, we observed that LPS increased P2X7-mediated HIF-1α expression in microglia, and inhibition of P2X7 led to reduced expression of HIF-1α and proinflammatory factors.

## Conclusion

We demonstrated the activation of microglia, the involvement of P2X7 and HIF-1α, and expression changes of cytokines in the MDH of the rat dental pulpitis model. Furthermore, our results revealed that downregulation of P2X7 alleviated dental pulpitis pain *via* reducing HIF-1α expression and decreasing IL-1β and IL-18 expression. Our findings reveal a new regulatory mechanism for dental pulpitis pain, which might contribute to the development of new therapies to relieve dental pulpitis pain.

## Data availability statement

The raw data supporting the conclusions of this article will be made available by the authors, without undue reservation.

## Ethics statement

The animal study was reviewed and approved by the Animal Use and Care Committee of the Fourth Military Medical University.

## Author contributions

LW and SS conceived and designed the experiments, provided financial support, and revised the manuscript. JZ and JS carried out the experiments, analyzed the data, and wrote the manuscript. RL and YL assisted JZ with histology and molecular biology experiments. HS, JZ, and JS assisted with animal studies. XL and JZ interpreted the data and revised the manuscript. All authors contributed to the article and approved the submitted version.

## Funding

This work was supported by the grants from the National Natural Science Foundation of China (81771095 and 82071235), Key R&D Program of Shaanxi Province (2017SF-103 and 2021KWZ-26), and State Key Laboratory of Military Stomatology (2020ZA01).

## Conflict of interest

The authors declare that the research was conducted in the absence of any commercial or financial relationships that could be construed as a potential conflict of interest.

## Publisher’s note

All claims expressed in this article are solely those of the authors and do not necessarily represent those of their affiliated organizations, or those of the publisher, the editors and the reviewers. Any product that may be evaluated in this article, or claim that may be made by its manufacturer, is not guaranteed or endorsed by the publisher.

## Supplementary material

Supplementary Figure 1 The knockdown efficiency of P2X7 siRNA. **(A,B)** Western blot analysis of the P2X7 after treatment with P2X7 siRNA. **(C)** The P2X7 mRNA levels was decreased after treatment with P2X7 siRNA.***P* < 0.01, ****P* < 0.001 versus NC group.
