# Supplementary figures and images for: Ligand-gated ion channel P2X7 regulates hypoxia-induced factor-1α mediated pain induced by dental pulpitis in the medullary dorsal horn
Source: Front Mol Neurosci. 2022 Oct 26;15:1015751. doi: 10.3389/fnmol.2022.1015751 (PMC9644926; doi:10.3389/fnmol.2022.1015751)

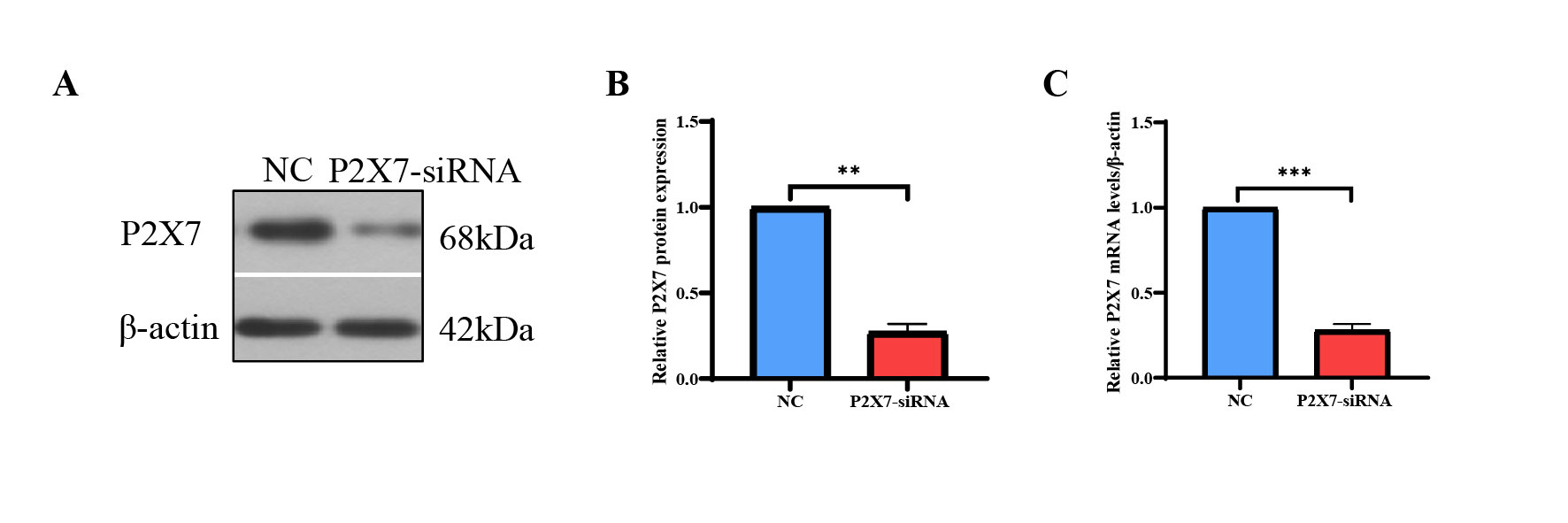

Supplement: Supplementary Figure 1 — The knockdown efficiency of P2X7 siRNA. (A,B) Western blot analysis of the P2X7 after treatment with P2X7 siRNA. (C) The P2X7 mRNA levels was decreased after treatment with P2X7 siRNA.**P < 0.01, ***P < 0.001 versus NC group. [file Image_1.JPEG]
